# Struggle for recognition, rights, and redistribution: Understanding the identity of parents of children with autism spectrum disorder in China

**DOI:** 10.3389/fpsyg.2022.981986

**Published:** 2023-01-10

**Authors:** Ying Liu, Karen R. Fisher

**Affiliations:** ^1^Department of Sociology, School of Humanities, Southeast University, Nanjing, China; ^2^Social Policy Research Centre, University of New South Wales, Sydney, NSW, Australia

**Keywords:** parent identity, families of children with autism spectrum disorders, China, qualitative, ethics of care

## Abstract

**Introduction:**

The number of children diagnosed with autism spectrum disorders (ASD) worldwide has increased rapidly in the past decade and China is no exception. Yet the identity development of Chinese parents of children with ASD is little understood. This study employed an ethics of care perspective to explore the identity of parents of children with ASD as shaped in their social–cultural context in mainland China.

**Methods:**

Qualitatively driven mixed-method design was adopted. Qualitative data about their experiences were obtained from in-depth interviews with 20 parents from 17 families of children with ASD in Beijing and participant observation of 9 participants’ daily parenting experience.

**Results:**

A complex and dynamic parenting identity was revealed. With limited recognition within and external to the family, parents experienced constant challenges toward their sense of self. The parents used strategies to assert their rights as carers and develop positive self-perceptions. Yet because of the unjust distribution of care work within families and with the state, the parents retained a sense of insecurity throughout the process of parenting. The parents’ sense of inferiority due to devaluing children with disabilities was accentuated by traditional Chinese cultural values about good parenting. They were intensely worried about the lack of policy for support as they and their children grew older.

**Discussion:**

The findings reinforce the need for recognition of parents’ dignity, capacity, and efforts in caring.

## Introduction

The number of children diagnosed with autism spectrum disorders (ASD) worldwide has increased rapidly in the past decade and China is no exception (Centers for Disease Control and Prevention, 2020). Many children with ASD need life-long support for therapy, education, employment, and independent living. Due to limited support services and particular cultural expectations, most care is from the children’s families (Tint and Weiss, 2016). ASD can add stress and additional challenges to family carers due to its complexity and invisibility (Gray, 2003; Kinnear et al., 2016; Chin et al., 2018; Hsiao, 2018). Although the rights of children with ASD have been gradually recognized, families are often marginalized in policy and services (McKenzie, 2016). Understanding their caring experiences is vital for improving the formal support available to families and children with ASD.

Parent identity is central to parents’ self-perceptions and parenting behavior (McBride et al., 2005; Hwang et al., 2020). Yet the identity of parents of children with ASD in non-western contexts remains under-researched. This study employed an ethics of care perspective to explore the identity of parents of children with ASD in mainland China. Analysis of the recognition, rights, and redistribution of care for children with ASD and their families can inform understanding about parents’ identity in their care work and the implications for support.

ASD is a neurodevelopmental disorder, characterized by differences in social communication, restricted and repetitive behavior, interests, and activities (Anderson et al., 2018; Tanu and Kakkar, 2019a, 2020). The latest report indicates about 1 in 44 children in the United States have been identified with ASD (Centers for Disease Control and Prevention, 2020). Parents of children with ASD can face many difficulties in their care responsibilities. Empirical studies reveal they have higher levels of stress, depression and anxiety compared with parents of children with other or no disabilities (Tint and Weiss, 2016; Chin et al., 2018; Hsiao, 2018; Tanu and Kakkar, 2019b). Sources of stress include the dissonance between their expectations and their child’s needs, the pressure to inform other family members, intensive contact with professionals, the financial as well as energy required to raise a child, and insufficient social support (Leiter et al., 2004; Hsiao, 2018). In addition, the common lack of visible disability combined with possible disruptive behaviors of children with ASD, means their parents are often subjected to stigma (Gray, 2003; Mak and Kwok, 2010; Kinnear et al., 2016). Most of the literature focuses on parents’ feelings of shock, denial, grief, chronic sorrow, and frustration (Shu et al., 2001), but several studies also report positive emotions of parents such as hope, love, strength, and joy (Corman, 2009; Corcoran et al., 2015; Lee et al., 2015; Kwok and Kwok, 2020). Chiaraluce (2018) indication that these complicated feelings could derive from their journey of redefining their self-identity. Chiaraluce’s study found that mothers’ experiences of marginalization challenged their self-identity, but they develop a sense of empowerment and control to balance their ruptured identities. Chiaraluce’s study indicates that care work affects the identity of parents. It also reveals that research about parents’ identity informs a comprehensive understanding about the contradictions and dynamics of parents’ experiences. Hence, identity of parents of children with ASD is a useful focus for research to inform practice. Parent identity consists of parents’ understandings of the meanings of being a parent, their expectations toward parenting, and their evaluation of self as a parent (Rane and McBride, 2000; Maurer et al., 2001). Empirical studies find that it plays a significant role in guiding the behavior of parenting (McBride et al., 2005; Hwang et al., 2020). The lives of parents of children with ASD are shaped by care they give, receive, and would prefer. Their parent identity is shaped by their experience of caring for their children with ASD and the care they receive as parents of children with disabilities.

Ethics of care theorists demonstrate that the inequalities experienced in care work influence the identity formation of formal and family carers (Lutz and Palenga-Mollenbeck, 2011; Shuttleworth, 2018; Akkan, 2020). They examine effect of the distribution of care responsibilities in public domain (Tronto, 2013). The ethics of care framework positions care as “not only the concern of those who are in some state of partial dependency but of society as a whole, all of who require and give care” (McKenzie, 2016). This acknowledgment of the inequalities of care responsibilities makes it a useful framework to research the identity of families of children with ASD. This application is especially relevant in the context of China, where policies about the distribution of care between state and citizen are rapidly changing. Williams (1999) and Williams (2010) developed an ethics of care in a framework of recognition, rights, and redistribution of care work as a way to construct an overarching frame for the social justice of care. This framework concerns “how rights to give and receive care with dignity are articulated and exercised, and how the resources necessary to support care are distributed” (Blaxland et al., 2018, p. 431). It is particularly useful in analyzing social policy and services of people with disabilities because of the multidirectional care relationships, the mix of informal and formal support, and the intersectional experiences of the disadvantaged (Kroger, 2009; Shuttleworth, 2018). According to Williams (2012), recognition of care includes visibility, voice, respect, and dignity of caregivers and care recipients. Rights include various aspects of human rights, including rights to receive financial and practical support as caregivers. Not only are goods and services distributed, but also responsibilities, power, and control. Instead of viewing care only as a responsibility within families, care can also be understood as a public good that requires resources. Therefore, an ethics of care demands that welfare systems recognize social injustice experienced in care work, assert people’s rights to give and receive care, and ensure people’s access to redistributed resources that make care available. Williams (2018) incorporated intersectional analysis in her framework to understand how multiple social relations at micro, meso, and macro levels frame care work. She emphasizes categories such as gender, class, institutions, and culture as relevant to analysis of differences and inequalities in care work.

Previous researchers reveal parent identity is situated in the social–cultural context of their community (Baumeister and Muraven, 1996). Chiaraluce’s analysis uncovers how family values shape the identity of mothers of children with ASD in the United States. Yet identity of parents of children with ASD in non-western countries is under-researched. The incidence of ASD is increasing rapidly in China, estimated at over 10 million people (Sun, 2019). Nationally, China adopts a human rights approach and has issued a series of policies that protect the therapy and education rights of children with ASD, e.g., “Law on the Protection of Persons with Disabilities” (first issued 1990), “Regulation on the Education of Disabled Persons” (first issued 1994), and “Regulation on the Prevention and Rehabilitation of Disabled Persons (issued 2017)” (Shang and Fisher, 2015; Zhao and Zhang, 2018). In 2017, the Chinese government issued two important disability policies. One policy is the revised “Regulation on the Education of Disabled Persons.” It states that general education to be the first and main choice for children with disabilities, which is different from the past special education priority principle (Zhao and Zhang, 2018). The other policy is the “Regulation on the Prevention and Rehabilitation of Disabled Persons.” It specifically requires local governments to provide financial, medical and facility supports to rehabilitation service for children with disabilities under 6 years old. Yet care responsibilities for children with disabilities still center on families. Families are often forced to take responsibility for supporting their child’s needs with only limited support from the government and community (McCabe, 2008). High levels of stress, depression, shame, and self-blaming are frequently reported in literature on Chinese parents (Chiu et al., 2013; Tang and Bie, 2016; Liu and Fisher, 2017; Chen et al., 2022). However, how Chinese parents constructed their identity when caring for children with ASD remains undiscovered. To fill in this research gap and better support Chinese families of children with ASD, an exploration of parents’ identity contents and development process within the Chinese context is needed.

In summary, this study adopts the ethics of care framework to understand how Chinese parents experience their identity as parents of children with ASD. Through analyzing the recognition, rights, and redistribution of parents’ care work for children with ASD, this study seeks to reveal the dynamics of parents’ identity in caring as shaped in the particular social–cultural context of mainland China and draw implications for disability policy and services.

## Materials and methods

The study adopted theory-driven qualitative analysis (Macfarlane and O’Reilly-de Brun, 2012) to explore the identity of parents. This approach was chosen to inform the design and analysis of the social phenomena through ethics of care theory so as to incorporate individual and structural elements. The fieldwork site was Beijing. It was the most suitable site because, as the capital, it is the center of research and practice of diagnosis, treatment, therapy, and education for children with ASD in mainland China. These resources have attracted families from around the country and with diverse characteristics. Ethical approval was gained from the Survey and Behavioral Research Ethics Committee of university.

### Participants

A purposeful sampling method was used to identify research participants (Coyne, 1997). The selection criterion was the parents had at least one child diagnosed with ASD. The sampling sought diversity in gender, family income, and family structure. Participants were recruited voluntarily through two NGOs that provided therapy services for children with ASD. The researchers collected and analyzed data after each participant was recruited. Recruitment continued until data saturation was reached when no new categories, themes, or explanations of parents’ identity development appeared in data of the 20th participant. In total, 20 parents (6 fathers and 14 mothers) from 17 families participated in the study. The children were aged 4–16 years old. Most of them were boys, consistent with the disproportionate diagnosis of ASD for boys. Three families had a low income and the rest were middle class. Most participants were married and two were single mothers. Four families also had other children without disabilities. The characteristics of participants are found in Tables 1, 2.

**Table 1:** Characteristics of participants.

Child’s name^*^	Gender	Age	Parent interviewed	Family income^**^	Siblings^***^
Anan	Boy	4 Diagnosed at 3.5 with mild ASD	Mother	Middle	-
Beibei	Boy	9 Diagnosed at 2 with severe ASD	Mother	Middle	-
			Father		
Cancan	Boy	6 Diagnosed at 3 with mild ASD	Mother	Middle	Yes
Dingding	Boy	10 Diagnosed at 2 with moderate ASD	Mother	Low	-
Enen	Girl	6 Diagnosed at 6 with mild ASD	Mother	Middle	-
Feife	Boy	11 Diagnosed at 3 with moderate ASD	Mother (single)	Low	-
Gege	Boy	9 Diagnosed at 2.5 with mild ASD	Father	Middle	-
Hehe	Boy	12 Diagnosed at 2 with mild ASD	Father	Middle	Yes
Jiejie	Girl	10 Diagnosed at 2 with mild ASD	Mother	Middle	-
Keke	Girl	8 Diagnosed at 2.5 with mild ASD	Mother	Low	-
Lele	Boy	9 Diagnosed at 2.5 with mild ASD	Mother (single)	Middle	-
Mengmeng	Boy	10 Diagnosed at 4.5 with mild ASD	Mother	Middle	-
			Father		
Nannan	Boy	13 Diagnosed at 3 with moderate ASD	Mother	Middle	-
Peipei	Girl	9 Diagnosed at 3.5 with moderate ASD	Mother	Middle	-
Qiuqiu	Boy	11 Diagnosed at 4 with moderate ASD	Father	Middle	Yes
Ranran	Boy	7 Diagnosed at 2 with mild ASD	Mother	Middle	-
Shasha	Girl	16 Diagnosed at 3 with moderate ASD	Mother	Middle	Yes
			Father		

Beijing interviews 2013–19. ^*^Pseudonym. ^**^Income information during family interview. ^***^No siblings with disabilities, 3 of 4 siblings were the second child.

**Table 2:** Demographic data of participants.

Characteristics	*N*
Gender	
Male	6 (30.00%)
Female	14 (70.00%)
Gender of participants’ children with ASD	
Boy	12 (70.59%)
Girl	5 (29.41%)
Age of participants’ children with ASD	
≤6	3 (17.65%)
6–12	12 (70.59%)
12–18	2 (11.76%)
Family income	
Low	3 (17.65%)
Middle	14 (82.35%)
Marriage status	
Married	15 (88.24%)
Divorced	2 (11.76%)
Siblings	
Yes	4 (23.53%)
No	13 (76.47%)

Twenty parents from 17 families, each family had one child with ASD.

### Data collection

The data were collected through participant observation and in-depth interviews. One researcher observed the daily parenting practice of 9 participants at the service from 2013 to 2014. Field notes were kept to record participants’ experience of giving and receiving care and their self-perceptions as parents. The context where participants were situated in was also described in field notes. The interview guide explored the content and development of parents’ identity through their experience around care. The questions were about parents’ experience of caring their children with ASD and engaging with social services for their children and themselves; their perceptions of self as parents of children with ASD; and the changes in their self-perceptions. The initial interview guide was revised during interviews with the first three participants. Follow-up interviews were conducted about missing information with these three participants after the researchers finalized the interview guide. One Mandarin-speaking researcher conducted all interviews in the parents’ first language.

Before data collection, the researcher explained the purpose, process, and outcomes of this study and highlighted that participants could withdraw at any time. Each participant signed an informed consent form. Two face-to-face interviews were conducted with most participants. The first round of interviews was conducted from 2013 to 2014. The second round of interviews was in 2019. The researcher asked participants about their experiences since policy changes in 2017–2018 about access to inclusive therapy and education in the second round of interview. A few participants, who did not answer all questions in the two interviews due to time limit or other reasons, had a third interview by telephone or online. Interview time for each participant ranged from 2 to 6 h. Most interviews were conducted at participants’ home. Three interviews were in one office of an NGO. All interviews were recorded with the permission of the participants.

### Data analysis

**Figure 1:**
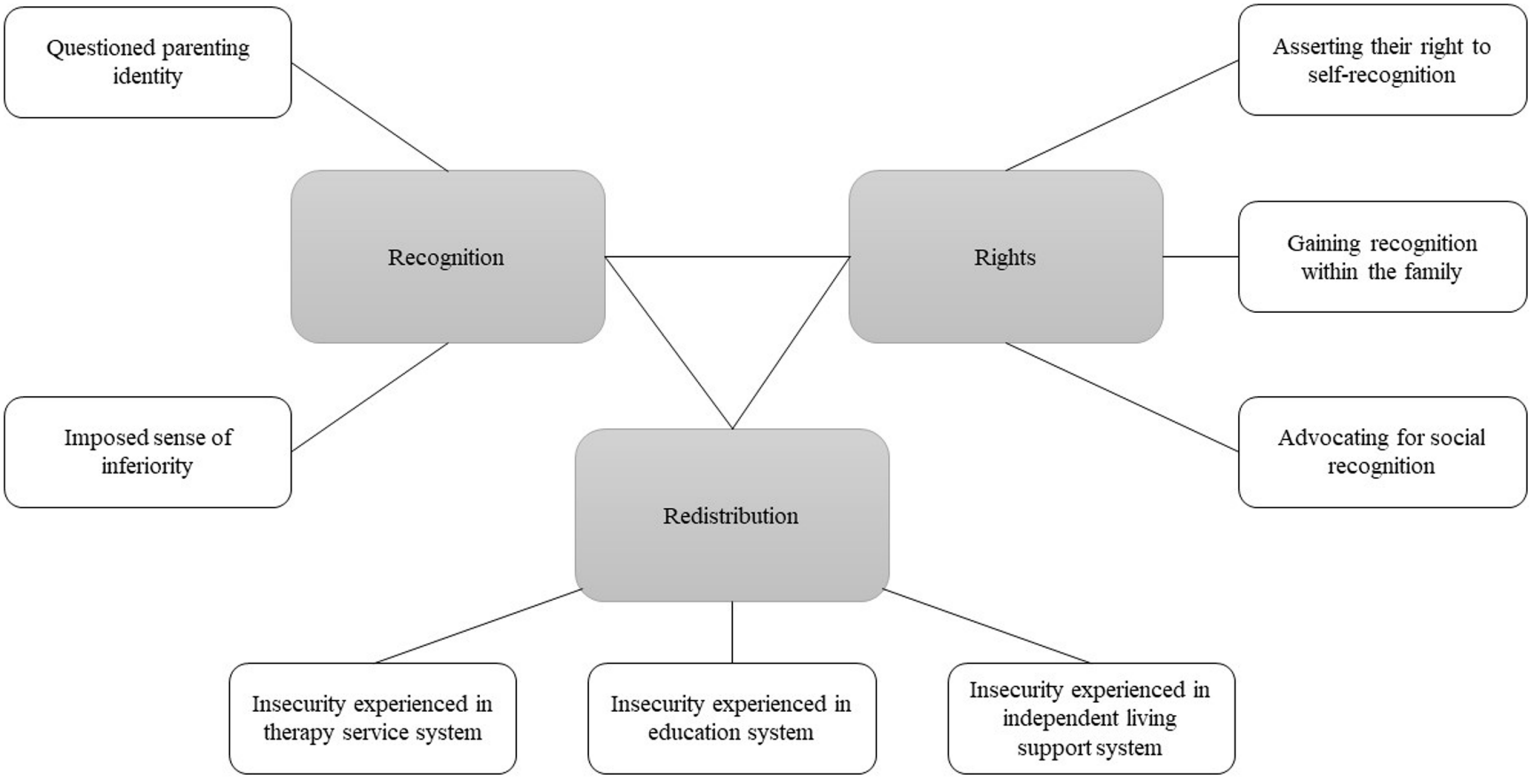
Thematic map.

Thematic analysis was used (Braun and Clarke, 2006). All interviews were transcribed verbatim. All parents and their children were assigned pseudonyms during data analysis to protect their privacy. Initial codes were generated about the parents’ caring experiences in their social–cultural context from interview transcripts and observation fields. For instance, “*Ranran’s diagnosis changed my life. I gave up my career. Ranran is now my career … I rarely have contact with previous friends or colleagues. I cannot stop thinking about how they would see me now. A loser who gave up her promising career to achieve nothing?*” was coded as “the sense of inferiority.” Examples of codes at this initial stage also included “bad perceptions toward self,” “misunderstandings from strangers” and “frustration when providing therapy to child.” The codes were collated into potential themes regarding participants’ understanding and expectations of themselves as parents of children with ASD and the changes in their perceptions. For example, codes such as “criticizing parents’ parenting style” and “complaining parents are irresponsible” were considered to be related and were grouped with codes such as “self-blaming raised from frustration during therapy.” These codes were collated into potential theme “questioned parenting ability.” These themes were reviewed, revised, and clustered into categories of the ethics of care framework. Recognition, rights, and redistribution of care work were the three main categories in the analysis. Sub-themes such as “asserting their right to self-recognition,” “gaining recognition within the family,” and “advocating for social recognition” were all about participants’ endeavor to protect their rights in care, thus were categorized into theme “Rights.” The thematic map of themes and sub-themes could be found in Figure 1. The quotes and themes were sent to participants for feedback and discussion about missing or additional data to ensure integrity of the analysis.

## Results

Three major themes emerged from findings about parents’ experiences that are related to the recognition, rights, and redistribution of caring for children with ASD. Within each theme, sub-themes are illustrated with representative quotes.

### Recognition—A constantly challenged self

While the parents devoted themselves to caring for their children with ASD, they felt that their parenting ability was not recognized. They said that their efforts and dignity as carers were not socially appreciated because children with ASD were devalued in Chinese society. Accordingly, constant challenges to their identity as competent parents stood out in the parents’ self-perceptions.

Since the diagnosis of Nannan, everything has changed. I used to be an excellent, confident and popular person … but now … every now and then, something would happen and make me feel bad about myself. (Nannan’s mother)

Two aspects of the constant challenges to the recognition of their parenting emerged, where the people around them questioned their parenting ability and imposed a sense of inferiority on them and their children.

#### Questioned parenting ability

Parents took the main responsibility for caring for their children with ASD, but they said their ability to parent was not always recognized. Many people attributed the children’s behaviors to the parents’ lack of capacity. Perhaps, the public judgment was due to their limited knowledge of ASD and the invisibility of the disabilities in the children’s appearance. Almost every participant said other people criticized them as irresponsible or incapable, sometimes even their own family members.

Once Cancan was screaming very loudly on the subway. I tried to stop him, but he just won’t listen to me. All the other passengers were staring at us. A few of them blamed me for Cancan’s misbehavior. (Cancan’s mother)

Of course, I have come across misunderstandings. I think every parent [of children with ASD] does. Even my own brother did not believe in me. He loves Anan. But he doesn’t understand ASD. He thinks that it’s my “over-protective” parenting that makes Anan too shy. (Anan’s mother)

Even if among people who were familiar with ASD, their parenting ability was not recognized. The parents gave the example of the corruption of the meaning of “parent is the best teacher of the child with ASD.” The concept was popular among the parents interviewed and therapists used it to promote parents’ participation in therapy. However, they said the way therapists used it was to assign the role of therapist onto parents, which resulted in an expectation of super-parents. Parents were expected to arrange and conduct therapy for their children. Accordingly, the service judged the parents by the outcome of their children. A few “successful” parents (whose children made very fast and obvious progress) received praise, but most parents did not receive recognition. They felt constantly frustrated, full of self-doubt and self-blame.

I devoted myself into learning and practising how to train Enen. But it’s hard to teach Enen and change her symptoms. It’s so frustrating. I felt like that I was a bad mother, who could not fulfil the obligation of training my own child. (Enen’s mother)

The experience of Enen’s mother was common among many participants. Keke’s mother said that she also felt disappointed in herself because she was not at the therapy her child needed.

In the first two years (of conducting therapy for Keke by myself), I was really upset. I kept questioning and criticizing myself for being a bad therapist. I was afraid that my incapability would hinder the rehabilitation of Keke. (Keke’s mother)

#### Imposed sense of inferiority

Parents also reported that other people imposed on them a sense of inferiority in their parenting. The social judgment reinforced the parents’ self-stigma about being inferior to other parents because their child had disabilities. Many parents admitted that they felt that their value as a parent was not socially recognized because their children had ASD. These parents’ self-image was closely bound with their children. In their communities, good parents have children with good academic performance at school, who enroll in top universities and find a good job to honor their parents. Many children with ASD cannot meet these expectations. Although the term ASD is more familiar in China now, respect was not widely extended to the children and parents in the study. Some parents were told by doctors that their children with ASD were “abnormal” and “hopeless.” Their children were not welcomed at their local schools and experiences of social discrimination and isolation were common. Consequently, parents felt that their efforts and dignity as carers were not socially recognized.

We parents are defined and judged by children’s performance and achievements. Even if you are not very successful, having an excellent child would make you a winner. But in my case, Mengmeng has ASD. No doubt I am a loser … Before, I was excellent, I was outstanding, but now, I feel like that my colleges, my friends, they are all better than me, because of Mengmeng. (Mengmeng’s father)

Once Beibei’s classmate asked me if Beibei was mentally retarded. It made me realize that no matter how hard I tried and how much progress Beibei made, he could never be like other children with ASD. He will always be abnormal in the eyes of other people. (Beibei’s father)

These statements were particularly common among the middle-income parents. These parents usually had good educational backgrounds and good jobs, which they were proud of. Many of them said that they had very high expectations toward themselves and their children. However, their child’s diagnosis of ASD had lowered their self-confidence.

Beibei’s father and I both come from small towns. We worked hard to acquire success in big cities. Our family, friends and colleagues all admire and respect us. Before [Beibei’s diagnosis], I thought that we were excellent. Our child should be excellent, too. I had hoped he would go to a famous university, get a PhD degree and become an excellent person. I did not expect that he could never reach even half of our expectations. His diagnosis was a huge blow to us. (Beibei’s mother)

It’s hard for me. We parents would expect our children to be more successful than us, or at least as good as us. I never thought my child could hardly be normal. It’s just too far away from my life plan. I won’t say it’s a nightmare. But it’s really hard for me to accept it. (Hehe’s father)

The mothers and fathers differed in some interpretations about recognition of their value as parents. The mothers usually felt more socially judged on their children’s achievements and behaviors compared with most of the fathers. Over half the mothers gave up their jobs and devoted themselves fully to parenting to better care for their child. It allowed them to concentrate on raising their child, but it also brought extra stress on their self-worth, which was now dependent on whether their child’s value could be socially recognized.

Ranran’s diagnosis changed my life. I gave up my career. Ranran is now my career … I rarely have contact with previous friends or colleagues. I cannot stop thinking about how they would see me now. A loser who gave up her promising career to achieve nothing? (Ranran’s mother)

The fathers spoke about the concept of *xuemai* (family line), which was not in the mothers’ narratives. Some fathers expressed frustration and disappointment about their broken family line as their child would not marry or have descendants.

I did think that my family line was broken. My parents were very disappointed. I myself was upset, too. In fact, that’s why we had a second child. To continue my xuemai. (Qiuqiu’s father)

Three families had a second child after the first child was diagnosed with ASD. The parents said their wish for a “normal” child was their main motivation for a second child and it improved their sense of self-worth.

To be honest with you, after the birth of Nannan’s little brother, I started to feel much better. I am back to a normal life. (Nannan’s mother)

Qiuqiu’s brother saved me to some extent. I know I should not say this, but he has brought hope. He makes me feel I could have my life back again. (Qiuqiu’s father)

However, the joy of having a second child was accompanied with struggle and guilt. For example, Shasha’s mother felt guilty about both her daughters as she could not fully concentrate on Shasha’s therapy and she had to save parenting time to spend with Shasha’s sister. She also worried whether Shasha’s sister would share caring responsibility for Shasha as she grew old enough and what effect that would have on Shasha.

While much of the community did not recognize the value of the parents and children with ASD, the parents formed their own community to look for mutual recognition and support. However, many of them felt that these peer communities were also marginalized. Several of them kept their “parent of children with ASD” as a secret identity. It indicated that they did not recognize their own dignity and value as a carer of a child with ASD.

I feel welcomed, accepted and comfortable here [with other parents of children with ASD]. But it’s still an abnormal community. (Enen’s mother)

I have to warn myself, not to be too happy in the community (of parents of children with ASD). I am afraid that such happiness would make me relax my concentration on Lele’s social inclusion. Afterall, Lele could not live in this small community forever. He will have to face the normal world someday, and I am responsible for that. (Lele’s mother)

In summary, parents took major responsibility for caring for their children with ASD, but their parenting ability was constantly questioned by the people around them. The devaluation of their children also imposed a sense of inferiority on the parents. Their efforts, ability, and dignity of care work were not sufficiently recognized, which constantly challenged their identity as parents. As a result, self-stigma was common among these parents.

### Rights—Developing positive self-perceptions

Despite the social judgments, parents asserted their right to recognition as carers of children with ASD. Through these attempts, parents were able to reconstruct their positive self-perceptions.

#### Asserting their right to self-recognition

The parents used various approaches to assert their right to self-recognition. Most parents learned to cherish each small gain they made in their child’s therapy and they regarded these small steps as recognition of their parenting capacity. Through this approach, parents were able to rebuild their confidence and feel more capable, irrespective of the lack to public recognition.

I felt really happy when Mengmeng made progress. His progress and improvement were like a reward for my endeavors. It indicated that I could be a trainer. I could train my child and I made something out of it. I became more confident of myself as a trainer and as a parent. (Mengmeng’s father)

Hehe’s progress is the best credit for me. Watching him getting better and better, I am feeling better about myself. (Hehe’s father)

The parents realized that their children’s improvements relied not only on their own care work, but also on the child’s support needs and access to social support. In these circumstances, many parents turned to acknowledge their parenting efforts and constructed their own meaning of good parenting of a child with ASD.

Every child was different. It makes no sense comparing the therapy outcomes of different children. Every parent is different, too. We parents have our own advantages and shortcomings. What really matters was whether parents tried their best. I’m not sure if I am a good mother. But I know I have tried my best. (Jiejie’s mother)

Now I have learned to let it go. I don’t blame myself anymore. I have my limits. Some things are just beyond my capacity. I choose to focus on those things that I am capable of. I think I have tried, tried hard. That’s enough for me. (Shasha’s father)

Most mothers also spoke about the quality of the relationship with their child and were very proud of the improvements the child had made. The emotional connections with their child brought self-recognition to these mothers.

Dingding and I have a very close relationship now. I can still remember when Dingding was 2 years old, he rarely responded to me. But things changed. I could see and feel Dingding’s emotional attachment with me. We are bonded. This makes me very happy. I feel that all my work on him has paid off. (Dingding’s mother)

Keke is strongly attached to me. I could feel it. She would be unhappy if I leave her for too long. She loves to hug me. And she won’t be mad at me even if I lose my temper with her. Her feedback comforts me. (Keke’s mother)

Paid work was an important source of self-recognition for fathers in this study. They described work as a critical opportunity for them to relax and concentrate on themselves. Over half of the fathers mentioned that they devoted more time to their paid work after the diagnosis of their children’s ASD. They regarded career success as a compensation of the loss in their family.

To be honest with you, I value my career more. I gain a sense of achievement from my career. Qiuqiu has ASD. I need to work harder and achieve more to make myself feel better. (Qiuqiu’s father)

In contrast to compensating for a sense of loss, some parents reported that they had grown personally through caring for their child with ASD, which also contributed to their self-recognition. Nearly half the parents suggested that caring for their child was an opportunity for them to develop a greater appreciation of life. Some parents even mentioned that they had become less judgmental and had learned to understand and care for other people.

Parenting Beibei was a different experience for me. It forced me to slow down. Living with Beibei taught me that every moment in life was important and precious. He also taught me to be grateful for what we have. He changed me a lot. (Beibei’s mother)

If Enen has no autism, I would be more judgmental. But what happens to Enen makes me realize that diversity is a good thing. It’s important to listen, to understand and to support. (Mengmeng‘s father)

#### Gaining recognition within the family

Recognition within the household and extended family was also key to changes in the parents’ identity. Most of the mothers said recognition from their husband was crucial to their wellbeing. Fathers’ acknowledgement of mothers’ parenting efforts, admiration of mothers’ parenting capacity and gratitude toward the mothers’ devotion into parenting reinforced a sense of appreciation and respect to the mothers.

Nannan’s father often talks about me in front of our family members and his friends. He tells these people that I am dedicated into caring for Nannan. He said that I am a good mother and Nannan could never have recovered so well without me. It feels really great when he does so. It’s wonderful that my husband could understand my devotion and appreciate my efforts. (Nannan’s mother)

I’m grateful that Dingding’s father always supports me. We don’t have much money. He could not afford expensive gifts for me. But he recognizes my efforts. He knows I’m doing these things for our family. That’s comforting. (Dingding’s mother)

An opposite case was Ranran’s family. Ranran’s father still denied Ranran’s disability and did not appreciate the efforts Ranran’s mother made. She defined his denial as her biggest source of stress and frustration.

Recognition from other family members also contributed to parents’ self-perceptions, particularly the attitude of grandparents. Shasha’s mother said that it took Shasha’s grandmother over 10 years to appreciate her devotion into Shasha. Though they did not live together, Shasha’s mother still felt upset and frustrated about it. Recently Shasha’s grandmother read Shasha’s mother’s book about her parenting journey with Shasha. She phoned Shasha’s mother and apologized for her misunderstanding. This call brought much comfort to Shasha’s mother as she felt she was finally recognized.

#### Advocating for social recognition

Several parents participated in advocacy activities to fight for social recognition of parents of children with ASD. Some parents, like Hehe’s father, advocated for policy to improve social welfare for children with ASD and their carers. Some of them, like Shasha’s mother, organized charity activities to promote recognition of children with ASD and elevate the children’s rights. Mengmeng’s mother also participated in online discussion to oppose stigma. Her online action arose when an education professor posted a blog article claiming that ASD was not a real disability but rather was a label assigned to children by their incapable parents. Many parents of children with ASD were angry about the article, so they flooded the blog as a spontaneous protest against the false statements about them. Although their advocacy efforts did not bring about much change, the parents cultivated a sense of self-competence through the process.

I had complicated feelings. I was still angry and disappointed about people [who believed the professor]. But at the same time, some part of me felt good [for advocating]. (Mengmeng’s mother)

In summary, parents asserted themselves inside and outside their families for their rights to self-recognition, recognition within their family, and social recognition as carers of children with ASD. Through their various strategies, parents made claims and called for appreciation and respect for their efforts, capacity, and dignity while providing care for their children.

### Redistribution—An enduring insecurity about their parenting

The parents’ sense of inferiority in their parenting and their effort to construct a positive self-image coexisted in the dynamic tensions within their self-identity. Whereas an expectation from the ethics of care framework would be that the welfare system supports families in their responsibility for care, the families were unhappy with the degree to which they were expected to provide the care alone. The parents reported that they carried the major responsibility for care, and the current welfare system failed to address their needs. Consequently, they had an enduring sense of insecurity about the adequacy of their parenting.

As the main carers, parents needed to not only take care of children’s daily life, but to also navigate the welfare system to find support for their child’s social inclusion. Parents said that current system was not supportive enough. Parents felt that they had to “fight against the world all by myself” (Anan’s mother). Three sub-themes emerged from parents’ description of their experienced insecurity when engaging different social service systems.

#### Insecurity experienced in therapy service system

Though the provision of therapy service for children with ASD was increasing, parents had difficulties identifying high-quality therapy service. Most participants had limited knowledge of ASD or therapy service when their child was diagnosed. Due to the lack of official guidance about quality therapy service, parents had to rely on their own judgments. Most families tried several times to find appropriate therapy services for their children. Nearly half of the participants said that the process of selecting therapy services was exhausting.

I was in a total mess at that time. I had never heard about ASD before. Suddenly my child was diagnosed with ASD and the doctor said he should receive therapy as soon as possible. There were only two or three famous therapy organizations, but they were all full. I had to find other organizations. But how to select the good ones? I tried many ways. I searched online, asked other parents and even visited the organizations myself. Even so, the first three organizations we tried were not satisfactory. The whole selecting process was exhausting. (Ranran’s mother)

I wish I had help at that time. I did not have a clue (on how to select a therapy service). I felt helpless. (Dingding’s mother)

#### Insecurity experienced in education system

Many parents of children at and under primary school age said that their child was not welcomed at their local school. The education policy guarantees enrolment for children with ASD into their local school, but most schools do not have an inclusive environment due to a lack of support resources. The parents had to negotiate with the school about educational arrangements. Sometimes they made extra efforts to create an inclusive opportunity for their child, such as building relationships with school teachers to gain their support. Yet many parents continued to receive regular complaints from school about their children’s behavior, which made them feel insecure.

Every time my phone rings, I am very nervous and anxious. I’m afraid it’s from Jiejie’s teacher, which means that I need to deal with trouble … I don’t know if he could stay in this local school. New problems appear. I don’t know if I have the capacity to solve every new problem in the future. Maybe someday we will have to leave this school. (Jiejie’s mother)

Peipei was not doing well in the school. The teachers were nice, but they did not have the energy or resource to look after Peipei. Several parents of Peipei’s classmates even complained to school that Peipei made trouble to his classmates and tried to ask us to leave. I said no to the headmaster. Peipei stayed. But I don’t know when this going to happen again. I’m not sure if I could stand my ground next time. (Peipei’s mother)

#### Insecurity experienced in independent living support system

The parents of older children were most anxious about the lack of independent living support for people with ASD. They worried about how their children would be supported when the parents were older or died. Some parents expected siblings of children with ASD or other family members to care for them in the future but they were uncomfortable with the implications of the responsibilities on their families. A few parents tried to find solutions to these long-term problems but gave up when the attempts were beyond their ability and resources. They could not stop worrying about their child’s future because they had no idea whether their child would be socially included and receive quality care as adults. As a result, a sense of insecurity was always hovering in their minds.

There’s no policy addressing this issue [of supported living for adults with ASD]. The biggest anxiety of us, of all parents is the worry about future. I choose to ignore this future issue so that I won’t be buried by worries and anxieties. But I know it’s there, waiting for me. We feel very insecure. (Hehe’s father)

What I worry most is how Feifei going to live when I am not available. I will get old and I will pass away. I could not image how he would be then. I try to not think about the future. But it comes to me at times. I don’t know what to do. (Feifei’s mother)

In summary, the parents felt that caring responsibilities were mostly shared within the children’s family and not well redistributed through policy or services. Their personal endeavors could not overcome the institutional barriers they faced or supplement the deficiencies they encountered in the welfare services available to them. Parents experienced difficulties when engaging social welfare system, which left the parents trapped in an enduring sense of insecurity.

## Discussion

This study contributes to current research and practice to support parents of children with ASD by revealing the complexities and tensions in parents’ identity as shaped in the social–cultural context of mainland China. The framework enabled an examination of the personal, family, and community elements of the parents’ identity, within the particular value, policy, and service context of mainland China. It reveals how the recognition, rights and redistribution of parents’ care work shape their identity development. The parents experienced constant challenges to their sense of self as good parents. Through various strategies, they sought to maintain positive self-perceptions. But they also dealt with underlying tensions within the family, community, and institutional structures that generated a pervasive sense of insecurity in their parenting identity.

The findings echo previous studies about the positive and negative elements of parents’ self-perceptions when parenting children with ASD (Corcoran et al., 2015; Lee et al., 2015; Kwok and Kwok, 2020; Chen et al., 2022). The current study further reinforced the dynamic tensions between these perceptions, which help policy and service practitioners to grasp a more complete understanding toward these parents. The implications from the focus on parents’ identity point toward the need for social recognition of parents’ caring efforts, capacity, and dignity when supporting children with ASD and their families. Policy should focus on reducing social stigma toward parents by increasing information about children with ASD and the caring experiences of their parents in local communities. Practice guidance could also encourage practitioners to adopt an empowering approach when working with parents, with a particular emphasis on the strengths and achievements of parents.

Parents were blamed by their family, other parents, and the general community for their incapacity to manage their children’s behavior and were looked down on for having a child with disabilities. The parents internalized these social judgments, which resulted in self-stigma about their inferiority as good parents. These experiences of stigma and blame are similar to studies with parents of children with ASD in western countries (Gray, 2003; Ryan and Runswick-Cole, 2008; Kinnear et al., 2016; Chiaraluce, 2018), and are accentuated by traditional Chinese cultural values of family and parenting. Chinese parents are confronted with more hostile social judgments and reported a stronger sense of inferiority than parents in western countries. Confucian cultural concepts of parenting relate children’s achievements to parents’ worth and children are deemed to represent the “report card” of parents (Ng et al., 2014). In addition, reproducing for maintaining the family bloodline is defined as one core obligation of filial piety (Shek, 2006). These cultural values might contribute to the heightened challenges to identity from parenting a child with ASD. Acknowledging the challenges to parents’ self-image and self-confidence implies that an empowering approach is highly suggested when working with parents.

An encouraging finding was that some of the Chinese parents, when faced with challenges, especially from the community or institutions, actively asserted their agency and negotiated to fulfill the rights of their children and themselves. They felt pride about their action, especially when it resulted in change. Chiaraluce (2018) found that families of children with ASD reframed their identity through redefining their role as caregivers and their participation in support networks. This study indicated parents developed positive self-perceptions from other resources, such as their close parent–child relationship, achievements from paid work, recognition from family members, and participation in advocacy. Unfortunately, current family education programs for children with ASD have been dominated by medical model of disability and reinforce the incapability of parents (Liu, 2016). The agency of these parents was ignored. It’s crucial for practitioners to realize the importance of maintaining positive self-perceptions for these parents. In future services for parents of children with ASD, practitioners should transform into strength-based perspective and value the agency of parents. Strategies adopted by parents to improve self-confidence and parenting efficacy at different levels could also inform practitioners the effective ways to promote parents’ self-perceptions.

The analysis also demonstrated the diversity of parents’ caring experience and needs. Understanding this diversity could help practitioners better support families of children with ASD. The findings extended Gray’s (2003) work about the gendered differences in coping strategies. Not only did career have special meaning for the fathers’ identity, but also recognition within the family was an important source of self-efficacy for the mothers. This finding suggests practitioners may use different strategies to empower mothers and fathers. Family income differences were also observed in the study. Middle income families generally had more support and more access to services, but these parents had stronger sense of inferiority in their parenting identity than low-income families. A possible explanation might be that middle income families were more inclined to compare children’s achievements, and they had higher expectations toward their children and themselves (Yang, 2006). Knowing that middle income families may experience additional pressure from self-evaluation, practitioners could give more concern about promoting self-confidence of these parents. Four families with siblings with no disabilities reported extra experience of comfort and guilt. Therefore, practitioners should pay special attention to parents’ emotional well-being and possible needs to balance family relationship when working with such families.

Moreover, the sense of enduring insecurity about the uncertain future of their children was particularly salient to Chinese parents of children with ASD. This might be explained by policy context in mainland China. The current welfare system focuses on financial support for children’s therapy and regulates equal access to education, but almost ignores the supported living and aging needs of adults with disabilities (Hao and Xu, 2015). The results of this study indicate that such assistance is far from enough, which affects parents’ identity as they struggle to fill the gap in responsibility toward their child. Families continue to be forced to take the primary responsibility for care and they worry about their children’s fate as the parents and children grow older. An ethics of care perspective highlights unjust distribution of caring responsibilities, with the implication that the state should provide funding and resources to ensure care is available and accessible (Williams, 2012). Therefore, practical support and resources are necessary for social inclusion of parents and children with ASD, particularly in living support for adults with ASD.

This study had several limitations. Since the participants were recruited through organizations that provide therapy for children with ASD, families without access to services were not included. Only three parents from low-income families participated, which may not have application to the experiences of other low-income families. In addition, two new policies regarding education and therapy rights of children with disabilities were introduced in 2017, after most of the interviews. This limitation was addressed with follow-up questions in 2018 and 2019, although all the changes from the new policies may not be captured in this study. The article is about the experience of parents in Beijing. The size and diversity of China means that results cannot be generalized to locations without similar characteristics.

Future research could further investigate the diverse experiences and unmet needs of different types of families, such as low-income families and families with siblings. Research about the impact of policy changes in the welfare system for children with ASD in mainland China and differences between locations could examine how these changes may shape parents’ caring experiences and identity. Children with ASD could also be included as research participants in future studies.

## Data availability statement

The datasets presented in this article are not readily available because some participants require that only the corresponding author could hear their interview tapes or see their interview transcripts. Requests to access the datasets should be directed to YL, liuyingsoc@seu.edu.cn.

## Ethics statement

The studies involving human participants were reviewed and approved by Survey and Behavioral Research Ethics Committee of The Chinese University of Hong Kong. The participants provided their written informed consent to participate in this study.

## Author contributions

YL: conceptualization, investigation, data analysis, and writing – original draft. KF: conceptualization, supervision, and writing – review and editing. All authors certify that they have participated sufficiently in the work to take public responsibility for the content, contributed to the article and approved the submitted version.

## Funding

This study is supported by “Fundamental Research Funds for the Central Universities” (2242022S20003). The funding covers the publication fee of this study.

## Conflict of interest

The authors declare that the research was conducted in the absence of any commercial or financial relationships that could be construed as a potential conflict of interest.

## Publisher’s note

All claims expressed in this article are solely those of the authors and do not necessarily represent those of their affiliated organizations, or those of the publisher, the editors and the reviewers. Any product that may be evaluated in this article, or claim that may be made by its manufacturer, is not guaranteed or endorsed by the publisher.
